# The Neglected C of Intercultural Relations. Cross-Cultural Adaptation Shapes Sojourner Representations of Locals

**DOI:** 10.3389/fpsyg.2021.611630

**Published:** 2021-03-23

**Authors:** Kinga Bierwiaczonek, Sven Waldzus, Karen van der Zee

**Affiliations:** ^1^Department of Psychology, University of Oslo, Oslo, Norway; ^2^Instituto Universitário de Lisboa (ISCTE-IUL), Centro de Investigação e Intervenção Social (CIS-IUL), Lisboa, Portugal; ^3^VU University Amsterdam, Amsterdam, Netherlands

**Keywords:** cross-cultural adaptation, outgroup representations, reverse correlation task, stereotype valence, intercultural relations

## Abstract

We investigated, by means of the Reverse Correlation Task (RCT), visual representations of the culturally dominating group of local people held by sojourners as a function of their degree of cross-cultural adaptation. In three studies, using three different methods (reduced RCT, full RCT, conceptual replication) with three independent samples of sojourners and seven independent samples of Portuguese and US-American raters, we gathered clear evidence that poor adaptation goes along with more negative representations of locals. This indicates that sojourner adaptation is reflected, at a social-cognitive level, in the valence of outgroup representations.

## Introduction

While the increasing cultural diversity of contemporary societies brings new opportunities for socio-cultural development, it also carries the risk of intergroup tensions. Hostile responses toward a perceived increase in cultural, ethnic, or other diversity can take various forms, from prejudice and discrimination (cf. Wright and Taylor, 2007) to radicalization and acts of violence (cf. Hafez and Mullins, 2015). As previous research shows, intergroup tension is reflected in people's visual representations of ethno-cultural outgroups. For instance, majority members who are highly prejudiced against immigrants visualize a prototypical face of this outgroup as criminal and untrustworthy (Dotsch et al., 2008).

An analogous phenomenon could be expected for minority members, for example sojourners who fail to adapt to the host culture. Sojourners with adaptation difficulties are known to perceive high intergroup tension (Wilson et al., 2013), and it seems reasonable to assume that such perceptions are partly reflected in social cognitions, that is, in negative representations of locals. The current set of studies investigates, by means of Reverse Correlation (Dotsch et al., 2008), visual representations of the cultural majority held by sojourners as a function of their degree of cross-cultural adaptation.

### Cross-Cultural Adaptation and Social Cognition

According to the ABC model of intercultural contact (Ward et al., 2001), adaptation occurs at three levels: Affect, Behavior, and (social) Cognition. In research practice, however, adaptation tends to be studied as bi-dimensional. The first dimension, psychological adaptation, is related to affect and refers to sojourner well-being; the second dimension, socio-cultural adaptation, is related to Behavior and refers to the quality of sojourner functioning within the host culture (Searle and Ward, 1990; Ward et al., 2001). Cognitive adaptation has received much less attention so far.

Most research on adaptation-related cognition has studied social identity shifts resulting from intercultural contact within the broader framework of acculturation research under the assumption that such identity shifts precede adaptation outcomes in a causal chain (cf., Berry, 1997; Ward et al., 2001; Ward and Geeraert, 2016). That is, the social-cognitive aspect of adaptation has been long considered as a part of the overall process rather than an outcome. In acculturation theory, this focus has recently shifted with Berry's (2015, 2017) addition of *intercultural adaptation*, a dimension that refers to “relating well” with other groups across cultural boundaries and covers social-cognitive outcomes such as “mutually positive ethnic attitudes and a lack of prejudice and discrimination” (Berry, 2017, p. 20). In acculturation research, some noteworthy although rather exceptional examples of research on social cognition aspects related to adaptation include studies by Tadmor et al. (2009), who argued that an increased cognitive complexity resulting from biculturalism may be adaptive, and studies by Stanciu and Vauclair (2018) and Stanciu et al. (2019), who proposed the stereotype accommodation hypothesis (i.e., that immigrants can incorporate the stereotypical beliefs learned in the host culture into preexisting stereotypes).

Despite these advances, social cognition still has not been systematically investigated as a distinct third dimension of cross-cultural adaptation. Yet, there are valid theoretical and empirical reasons for doing so. With our research, we intend to fill this gap in the literature by systematically examining how sojourners' representations of a typical local person, potentially reflecting the social-cognitive aspect of adaptation, are empirically interrelated with affective and behavioral adaptation.

International transitions usually imply entering a social reality dominated by the cultural outgroup, the local people. Intergroup phenomena such as perceived discrimination (*r* = −0.50, the strongest effect in the meta-analysis by Wilson et al., 2013; and *r* = −0.41, one of the strongest effects in the meta-analysis by Bierwiaczonek, 2018) have an impact on cross-cultural adaptation, and our expectation is that this impact is partly reflected in social cognitions, that is, in negative outgroup representations. We understand these representations as the visual encoding of an overall negative stereotype (cf., Dotsch et al., 2008).

Theoretically speaking, there are several reasons why psychological and socio-cultural adaptation should be reflected at the social-cognitive level. First, negative stereotypes go in line with negative expectations concerning the behavior of the local people, which generates intergroup threat and intergroup anxiety; these, in turn, translate into negative emotions and stress (Stephan and Stephan, 1996; Riek et al., 2006), that is, undermine psychological and socio-cultural adaptation (Bierwiaczonek et al., 2017). Second, low levels of socio-cultural adaptation are characterized by uncertainty how to behave and unawareness of cultural constraints of local people's behaviors. Uncertainty contributes to intergroup anxiety and to feelings of threat (Stephan and Stephan, 1996; Riek et al., 2006). Unawareness increases the likelihood of attributing behaviors of locals to their alleged negative characteristics (Gilbert and Malone, 1995; Gawronski, 2004). Such correspondence bias may then be generalized to the entire host-national group, contributing to a negative representation (Mackie et al., 1996).

Finally, there is evidence to suggest that poor psychological and socio-cultural adaptation and negative cognitions may be products of the same relevant context conditions, such as low quality of intergroup relations. Negative contact experiences may both undermine psychological and socio-cultural adaptation (Bierwiaczonek, 2018) and contribute to negative attitudes toward locals (Barlow et al., 2012), possibly translating into a negative representation. At the same time, holding negative representations of and expectations toward locals can be considered appropriate if an intergroup relation is perceived as hostile, abusive, or conflictual (Jasinskaja-Lahti et al., 2009).

Conversely, the better the adaptation, the higher the awareness of the local culture and sojourners' capacity to cope with it (Ward et al., 2001). Higher awareness may add complexity to preexisting representations of locals; reduce uncertainty, intergroup anxiety, and threat (Stephan and Stephan, 1996; Riek et al., 2006); and decrease the probability of attributing negative traits through correspondence bias (Gilbert and Malone, 1995; Gawronski, 2004). All of these should result in more positive representations of locals.

In sum, there are several plausible reasons to predict a link between sojourners' adaptation and their representations of locals, yet this relation has not been studied so far in an unobtrusive way. Since the Affective, Behavioral, and Cognitive levels of adaptation are hypothesized to be interrelated, we expect that psychological and socio-cultural adaptation correlates positively with the valence of visual representations of locals held by sojourners (*H1*).

### The Current Studies

In a set of three studies, we examined sojourner representations of locals by means of the Reverse Correlation Task (RCT; Dotsch et al., 2008; Dotsch and Todorov, 2012). This task was considered optimal for our purposes because it allows for tapping into visual representations of any social group of interest while avoiding social desirability. Specifically, participants are requested to reproduce a prototype of a social group by repeatedly choosing between stimuli consisting of face images. Since the stimuli do not carry any explicit valence, by doing so, participants do not need to voice any feelings or opinions that could potentially go against social desirability. Individual responses to one trial are not interpretable, and it is only by combining a number of trials into one image that researchers can obtain the approximate visual representation of the outgroup at stake. The attributes of this representation are then explored using diverse techniques. RCT was previously used to grasp visual representations of ethno-cultural minority outgroups such as immigrants, as well as intergroup phenomena such as prejudice, showing that intergroup attitudes are reflected in the valence of outgroup representations (Dotsch et al., 2008; Imhoff et al., 2011). In our studies, we applied a two-phase variant of the RCT (Dotsch et al., 2008). In Phase I, three groups of participants with low, moderate, and high adaptation created one image per group, partly translating their representations of the local people. In Phase II, those images were evaluated both objectively, by means of pixel correlations, and intersubjectively, by independent raters, to find to what extent they differ in their valence accordingly to *H1*. In the below sections, we report all measures, manipulations, and exclusions in our studies.

## Study 1

### Methods in Phase I: Creation of Classification Images

#### Sample and Procedure

Emails requesting assistance with the recruitment of participants were sent to the International Offices of seven Portuguese universities who forwarded a link to our online survey to international students. Out of 160 started surveys, 122 were completed, resulting in a dropout rate of 24%, which is relatively low for online studies (Galesic, 2006). Four other participants were dropped because their adaptation scores were missing. The final sample consisted of 118 international students residing in Portugal (31.4% male, mean age: 25.6 years, 89% sojourning in Portugal for 12 months or less; most represented home countries: 13.6% Brazil, 11.9% Italy, 9.3% Poland, 32 other countries, each of them accounting for <5% of the sample).

#### Reverse Correlation Task

We followed the RCT procedure developed by Dotsch et al. (2008). However, while in a regular RCT participants usually perform 300–770 trials (cf. Dotsch et al., 2008; Imhoff et al., 2011; Dotsch and Todorov, 2012), in our study this number was reduced to minimize non-compliance with task instructions due to the repetitive and demanding features of the task, which may be problematic in online RCT studies given the absence of participant monitoring. Other reasons to reduce the number of trials may include resource constraints in terms of experimental time and/or costs of implementation.

Each participant was presented a randomized set of 50 trials out of a pool of 300 trials. All stimuli consisted of face images that were built of the same base face with random noise superposed. The base face was a morph of photographs of male faces taken in Lisbon as part of the artistic project the Face of Tomorrow (Mike, 2003). Morphs from this project were previously used in RCT studies (see Imhoff et al., 2011; Imhoff and Dotsch, 2013). Each trial consisted of a pair of face images with noise patterns consisting of pixels with opposite luminance values. The two face images were presented side by side, and participants were instructed to choose the one that looked more like a typical Portuguese person. One stimulus face consisted of the base face superimposed with a random noise pattern. The other was the base face superimposed with the negative of the same noise pattern (for technical details, see Dotsch et al., 2008; Dotsch and Todorov, 2012). The RCT was followed by adaptation measures and sociodemographic questions.

#### Cross-Cultural Adaptation

Cross-cultural adaptation was measured by the Brief Psychological Adaptation Scale (BPAS; Demes and Geeraert, 2014) and the Socio-cultural Adaptation Scale (SCAS; Ward and Kennedy, 1999), using five-point Likert scales for both instruments.

*BPAS* (8-items) is a measure of psychological outcomes specific for the cross-cultural context. It has been validated on a large sample of sojourners (*N* = 1,929) and shown to correlate in expected directions with constructs typically used in adaptation research to operationalize psychological adaptation (stress, anxiety, self-esteem, and satisfaction with life; see Demes and Geeraert, 2014). Sample items are as follows: “In the last 2 weeks, how often have you felt excited about being in your host country?” (+) and “In the last 2 weeks, how often have you felt out of place, like you don't fit into the host country's culture?” (−). Cronbach's alphas were 0.80 in the current study, 0.84 in Study 2, and 0.62 in Study 3^1^.

*SCAS* (17 items in this study) has been widely used in adaptation research and validated in various sojourner samples (see Wilson et al., 2013, for a review). Participants were asked how difficult it was for them to deal with everyday matters in the host country (e.g., “Making friends,” “Getting used to the pace of life”). Reversed coding was used so that higher scores indicated better adaptation. Cronbach's alphas were 0.86 in the current study, 0.78 in Study 2, and 0.71 in Study 3.

**Figure 1:**
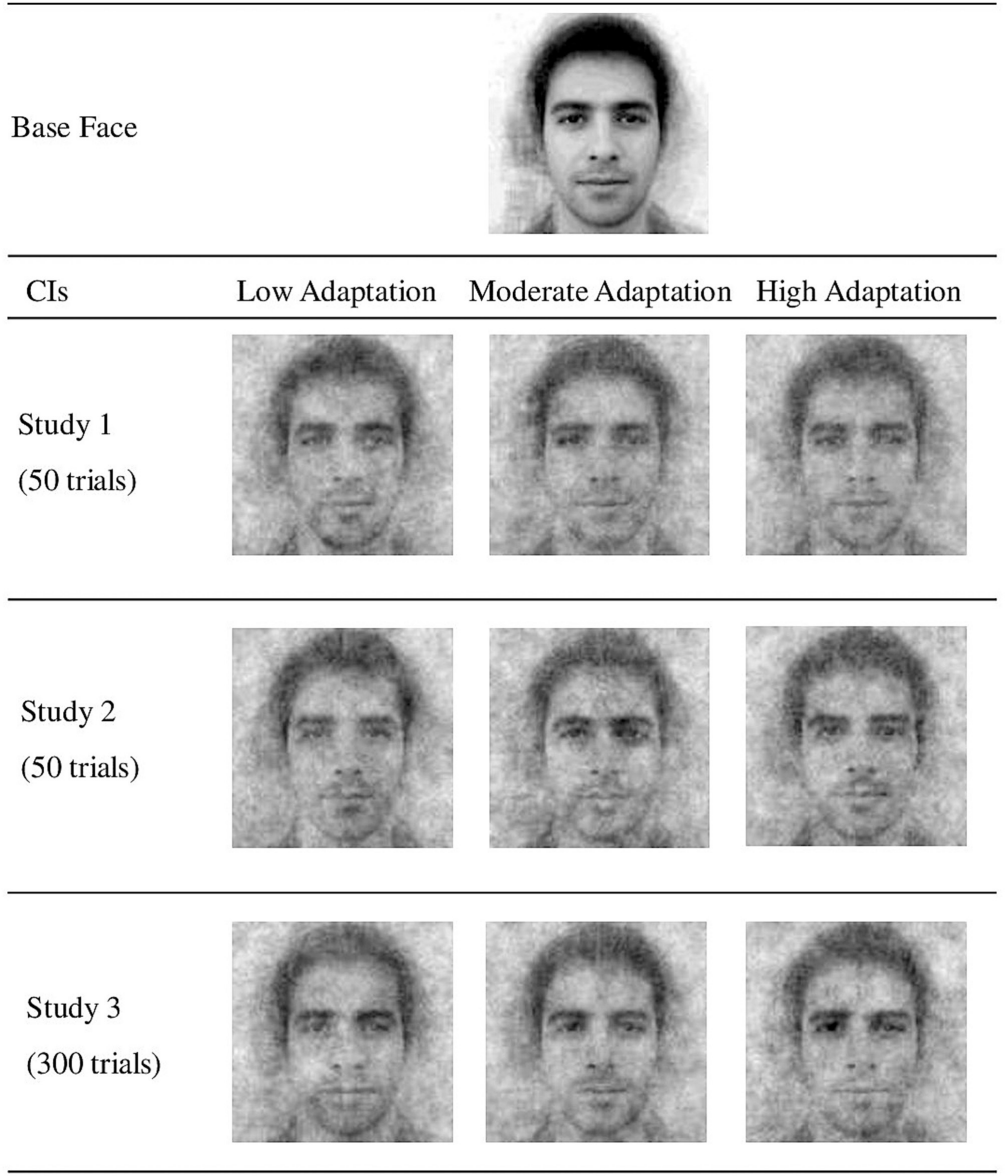
Classification images per adaptation level obtained in Studies 1–3.

In line with the ABC model (Ward et al., 2001), the two scales were strongly correlated in all three studies (0.48, 0.58, 0.59; all *p*s < 0.01). To obtain participants' overall cultural adaptation scores, we averaged scores on both scales to ensure that both scales have equal weight. Afterward, the sample was split on the 33rd and 66th percentiles into three groups: low adaptation (*N* = 39, *M* = 3.00, *SD* = 0.34), moderate adaptation (*N* = 39, *M* = 3.69, *SD* = 0.13), and high adaptation (*N* = 40, *M* = 4.21, *SD* = 0.19). We computed three Classification Images (CIs) by averaging all images chosen by all participants within each of these three groups (see Figure 1) using the R package *rcicr* 0.3.0 (Dotsch, 2015; in Studies 2 and 3, *rcicr* v. 3.4.1 was used). The three CIs were evaluated in Phase II.

#### Sociodemographic Variables

The survey included questions about participants' age, gender, home country, host university, length of stay in Portugal, and the amount of contact with local people inside and outside of the university.

### Methods in Phase II: CI Evaluation

#### Objective Evaluation

The CIs obtained in Phase I were evaluated in two ways: objectively, by assessing physical similarities between each pair of CIs, and intersubjectively, by submitting the CIs to the evaluation by three independent samples of raters. For the objective evaluation, we adapted the R code from Oliveira et al. (2019) to calculate the correlations between pixel luminance values of each pair of CIs: low with moderate-adaptation CI, low with high-adaptation CI, and moderate with high-adaptation CI^2^. Positive correlations indicate that two CIs are physically similar (i.e., the darker the pixels in a specific face region in one image, the darker the pixels in the same face region in the other image). Negative correlations indicate that two CIs are opposite (i.e., the darker the pixels in one image, the lighter on the other image). Null correlations indicate that the images share no similarities. Because the participants were instructed to recreate the prototype of the same ethnic group (the Portuguese), we expected some similarities (i.e., positive correlations) between all CIs. However, if the level of adaptation indeed differentiated these CIs, the correlations between the low-adaptation CI and the high-adaptation CI should be substantially smaller than between the remaining pairs of images.

#### Intersubjective Evaluation

While the procedure developed by Dotsch et al. (2008) only includes one evaluation, in our case the first evaluation gave unexpected results which, we assumed, had to do with the fact that raters were members of the RCT target population (Portuguese). Therefore, we recurred to two other independent rater samples: American raters (unrelated to the RCT target population) and Portuguese raters (to test whether the unexpected results were indeed due to nationality; see Appendix C in the Supplementary Materials, for the full rationale). None of the rater samples was informed that the CIs represented “typical Portuguese” faces as seen by sojourners. Raters were simply informed that they were participating in a study that examines “people's representations of others” and that they would be requested to evaluate images of human faces, with no further explanations. The CIs were presented to each rater in randomized order.

Power analysis was conducted in G^*^Power 3.1 (Faul et al., 2007) for a repeated-measures ANOVA with the following parameters: power 0.90, small effect size (*f* = 0.20, corresponding with $\eta_{\text{partial}}^{2}$ = 0.04), and moderate correlation between repeated measures (*r* = 0.50), establishing the optimal sample size of 55. First, 50 Portuguese students (28% male, mean age: 26.22 years, mostly students of psychology-−64%) evaluated the CIs in an online survey in Portuguese.

In a within-subject design, participants were requested to rate each CI on 1–10 continuous scales (sliders) on a set of 12 theoretically derived adjectives which tapped into the two hypothetical dimensions of stereotype content, that is, warmth (Trustworthy, Helpful, Friendly, Sociable) and competence (Intelligent, Competent; cf. Cuddy et al., 2008), as well as adjectives considered relevant for sojourner adaptation either as translating the pull toward the host national outgroup (Interesting, Attractive) or as related to potential intergroup tensions (Tolerant, Closed-Minded, Aggressive, Dangerous). Exploratory factor analyses with principal axis factoring and oblimin rotation conducted separately for ratings of each CI tended to extract, in seven analyses out of nine, two different factors: positive adjectives and negative adjectives (for details, see Appendix A in the Supplementary Materials). In Studies 1–3, correlations between these factors (calculated separately for the low, moderate, and high-adaptation CIs and for each rater sample) ranged from 0.03 to −0.49. We calculated composite scores for these two factors by averaging, separately, scores on positive (Cronbach's α range for low, moderate, and high-adaptation CIs across the three studies reported in this paper: 0.92–0.96) and on the negative adjectives (α range: 0.78–0.94).

Additionally, raters were shown the three CIs side by side and responded to three forced-choice questions: “If you had to choose one of these three people, who would you choose to…” (a) “…share your room in campus or a student flat with,” (b) “…carry out some university work with,” and (c) “…go to the cinema or a party with.” Raters also responded to questions about their age, gender, study domain, and whether they were of Portuguese nationality.

Second, the CIs obtained in Phase I were reevaluated by 50 American raters recruited via Amazon's Mechanical Turk (62% male, mean age: 31.5 years) in an online survey. We employed English versions of the items used in the first evaluation, adapted to a non-academic context whenever necessary (e.g., “Who would you choose to be your neighbor” instead of “share your room in campus”). We also included an additional forced-choice question related to intergroup threat: “Which person you would never want to meet in a dark empty street at night?”

Third, the CIs were reevaluated by a different, independent sample of 50 Portuguese students (46.3% male, mean age: 20.6 years, most represented study domains: psychology 31.5%, management 24.2%). We used the same online survey as in the first evaluation, but we added two more dimensions to grasp identity-related aspects (“Similar to yourself”; “Similar to a typical Portuguese”; see Appendix C in the Supplementary Materials for the rationale), and the forced-choice question related to intergroup threat: “Which person you would never want to meet in a dark empty street at night?”

### Results

#### Objective Evaluation

As expected, the low-adaptation CI showed a close to zero correlation with the high adaptation CI (*r* = 0.03, 95% CI [0.024, 0.031]), as well as with the moderate-adaptation CI (*r* = −0.05, 95% CI [−0.055, −0.047]), suggesting that there was no physical similarity between the low-adaptation CI and the remaining two images. In contrast to that, the moderate-adaptation CI was weakly positively correlated with high-adaptation CI (*r* = 0.24, 95% CI [0.236, 0.243]). Please note that, since the pixel was the unit of analysis here (*N* = 262,143), the *p*-values are not informative (all *p*s < 0.001).

#### Intersubjective Evaluation

To analyze the intersubjective evaluation data, we conducted repeated-measures ANOVAs separately for the two composites of positive adjectives and negative adjectives, as well as for each individual adjective on which the CIs were rated. The results for composite measures are reported in Table 1. The results for individual adjectives can be found in the Appendix B (Supplementary Materials). Moreover, we used the chi-squared test to check for differences in frequencies of choosing each CI in the forced-choice questions. The results of these analyses are reported in Table 2.

**Table 1:** Evaluations of classification images on positive and negative adjectives across studies 1–3.

	**Positive adjectives**						**Negative adjectives**					
	***M*_low_**	***SE*_low_**	***M*_mod_**	***SE*_mod_**	***M*_high_**	***SE*_high_**	***M*_low_**	***SE*_low_**	***M*_mod_**	***SE*_mod_**	***M*_high_**	***SE*_high_**
**STUDY 1**												
Evaluation 1 (PT)	4.37	0.25	4.95	0.24	4.67	0.26	3.08	0.31	2.54	0.27	2.98	0.28
	*F*_(2,96)_ = 4.01, *p* = 0.02, $\eta_{\text{p}}^{2}$ = 0.08, η^2^ = 0.02						*F*_(2,96)_ = 2.40, *p* = 0.10, $\eta_{\text{p}}^{2}$ = 0.05, η^2^ = 0.01					
	Quadratic contrast: *F*_(1,48)_ = 7.96, *p* = 0.01, $\eta_{\text{p}}^{2}$ = 0.14						Quadratic contrast: *F*_(1,48)_ = 4.20, *p* = 0.05, $\eta_{\text{p}}^{2}$ = 0.08					
	Linear contrast: *F*_(1,48)_ = 1.74, *p* = 0.19, $\eta_{\text{p}}^{2}$ = 0.04						Linear contrast: *F*_(1,48)_ = 0.17, *p* = 0.68, $\eta_{\text{p}}^{2}$ = 0.00					
Evaluation 2 (US)	5.74	0.21	5.92	0.21	5.87	0.23	3.55	0.27	3.05	0.29	3.03	0.27
	*F*_(2,98)_ = 0.52, *p* = 0.60, $\eta_{\text{p}}^{2}$ = 0.01, η^2^ = 0.00						*F*_(2,98)_ = 3.55, *p* = 0.03, $\eta_{\text{p}}^{2}$ = 0.06, η^2^ = 0.07					
	Quadratic contrast: *F*_(1,49)_ = 0.68, *p* = 0.41, $\eta_{\text{p}}^{2}$ = 0.01						Quadratic contrast: *F*_(1,49)_ = 1.75, *p* = 0.19, $\eta_{\text{p}}^{2}$ = 0.03					
	Linear contrast: *F*_(1,49)_ = 0.41, *p* = 0.53, $\eta_{\text{p}}^{2}$ = 0.01						Linear contrast: *F*_(1,49)_ = 5.02, *p* = 0.03, $\eta_{\text{p}}^{2}$ = 0.09					
Evaluation 3 (PT)	4.03	0.25	4.73	0.29	4.43	0.27	3.07	0.26	2.44	0.28	2.80	0.26
	*F*_(2,102)_ = 7.35, *p* = 0.00, $\eta_{\text{p}}^{2}$ = 0.13, η^2^ = 0.02						Greenhouse–Geisser *F*_(1.56, 78.14)_ = 4.35, *p* = 0.02, $\eta_{\text{p}}^{2}$ = 0.08, η^2^ = 0.02					
	Quadratic contrast: *F*_(1,51)_ = 9.90, *p* < 0.001, $\eta_{\text{p}}^{2}$ = 0.16						Quadratic contrast: *F*_(1,50)_ = 5.84, *p* = 0.02, $\eta_{\text{p}}^{2}$ = 0.11					
	Linear contrast: *F*_(1,51)_ = 4.81, *p* = 0.03, $\eta_{\text{p}}^{2}$ = 0.09						Linear contrast: *F*_(1,50)_ = 1.99, *p* = 0.16, $\eta_{\text{p}}^{2}$ = 0.04					
**STUDY 2**												
Evaluation 1 (PT)	4.08	0.25	4.10	0.22	4.58	0.27	3.04	0.29	3.37	0.33	2.26	0.24
	*F*_(2,88)_ = 5.01, *p* = 0.01, $\eta_{\text{p}}^{2}$ = 0.10, η^2^ = 0.02						*F*_(2,86)_ = 2.63, *p* = 0.08, $\eta_{\text{p}}^{2}$ = 0.06, η^2^ = 0.02					
	Quadratic contrast: *F*_(1,44)_ = 1.88, *p* = 0.18, $\eta_{\text{p}}^{2}$ = 0.04						Quadratic contrast: *F*_(1,43)_ = 3.33, *p* = 0.07, $\eta_{\text{p}}^{2}$ = 0.07					
	Linear contrast: *F*_(1,44)_ = 9.31, *p* < 0.001, $\eta_{\text{p}}^{2}$ = 0.18						Linear contrast: *F*_(1,43)_ = 1.75, *p* = 0.19, $\eta_{\text{p}}^{2}$ = 0.04					
Evaluation 2 (US)	5.19	0.22	5.09	0.22	5.79	0.20	3.87	0.26	4.15	0.29	3.24	0.22
	*F*_(2,108)_ = 5.64, *p* = 0.005, $\eta_{\text{p}}^{2}$ = 0.10, η^2^ = 0.04						Greenhouse-Geisser *F*_(1.75,94.71)_ = 4.31, *p* = 0.02, $\eta_{\text{p}}^{2}$ = 0.07, η^2^ = 0.04					
	Quadratic contrast: *F*_(1,54)_ = 3.54, *p* = 0.06, $\eta_{\text{p}}^{2}$ = 0.06						Quadratic contrast: *F*_(1,54)_ = 3.38, *p* = 0.07, $\eta_{\text{p}}^{2}$ = 0.06					
	Linear contrast: *F*_(1,54)_ = 8.70, *p* = 0.01, $\eta_{\text{p}}^{2}$ = 0.14						Linear contrast: *F*_(1,54)_ = 6.34, *p* = 0.02, $\eta_{\text{p}}^{2}$ = 0.11					
**STUDY 3**												
Evaluation 1 (PT)	3.56	0.24	4.03	0.23	4.48	0.23	3.67	0.27	3.35	0.25	2.89	0.29
	*F*_(2,90)_ = 9.36, *p* < 0.001, $\eta_{\text{p}}^{2}$ = 0.17, η^2^ = 0.06						*F*_(2,84)_ = 3.79, *p* = 0.03, $\eta_{\text{p}}^{2}$ = 0.08, η^2^ = 0.03					
	Quadratic contrast: *F*_(1,45)_ = 0.01, *p* = 0.93, $\eta_{\text{p}}^{2}$ = 0.00						Quadratic contrast: *F*_(1,42)_ = 0.07, *p* = 0.79, $\eta_{\text{p}}^{2}$ = 0.00					
	Linear contrast: *F*_(1,45)_ = 15.71, *p* < 0.001, $\eta_{\text{p}}^{2}$ = 0.26						Linear contrast: *F*_(1,42)_ = 7.20, *p* = 0.01, $\eta_{\text{p}}^{2}$ = 0.15					
Evaluation 2 (US)	4.67	0.22	5.21	0.22	5.45	0.22	4.24	0.24	3.83	0.25	3.40	0.23
	*F*_(2,106)_ = 9.52, *p* < 0.001, η^2^ = 0.04						*F*_(2,106)_ = 6.01, *p* = 0.00, $\eta_{\text{p}}^{2}$ = 0.10, η^2^ = 0.04					
	Quadratic contrast: *F*_(1,53)_ = 0.97, *p* = 0.33, $\eta_{\text{p}}^{2}$ = 0.02						Quadratic contrast: *F*_(1,53)_ = 5.01, *p* = 0.97, $\eta_{\text{p}}^{2}$ = 0.00					
	Linear contrast: *F*_(1,53)_ = 16.65, *p* < 0.001, $\eta_{\text{p}}^{2}$ = 0.24						Linear contrast: *F*_(1,53)_ = 0.00, *p* < 0.001, $\eta_{\text{p}}^{2}$ = 0.17					

*Mean evaluations and standard errors of Classification Images (CIs) obtained in the three studies are reported. Indexes _low,mod,high_ refer to mean evaluations of the low-adaptation CI, moderate-adaptation CI, and high-adaptation CI, respectively*.

**Table 2:** Frequencies of choosing Classification Images (CIs) in forced-choice questions across studies 1–3.

	**Cinema**			**Work**			**Neighbor/roommate**			**Dark street**		
	**Low**	**Mod**	**High**	**Low**	**Mod**	**High**	**Low**	**Mod**	**High**	**Low**	**Mod**	**High**
**STUDY 1**												
Evaluation 1 (PT)	15	17	17	13	15	21	11	17	21	–	–	–
	$X_{\left(2\right)}^{2}$ = 0.16, *p* = 0.92			$X_{\left(2\right)}^{2}$ = 2.12, *p* = 0.35			$X_{\left(2\right)}^{2}$ = 3.10, *p* = 0.21					
Evaluation 2 (US)	9	20	21	10	19	21	10	18	22	29	15	6
	$X_{\left(2\right)}^{2}$ = 5.32, *p* = 0.07			$X_{\left(2\right)}^{2}$ = 4.12, *p* = 0.13			$X_{\left(2\right)}^{2}$ = 4.48, *p* = 0.11			$X_{\left(2\right)}^{2}$ = 16.12, *p* < 0.001		
Evaluation 3 (PT)	10	25	19	6	23	25	6	21	27	39	11	4
	$X_{\left(2\right)}^{2}$ = 6.33, *p* = 0.04			$X_{\left(2\right)}^{2}$ = 12.11, *p* = 0.002			$X_{\left(2\right)}^{2}$ = 13.00, *p* = 0.002			$X_{\left(2\right)}^{2}$ = 38.11, *p* < 0.001		
**STUDY 2**												
Evaluation 1 (PT)	9	9	27	5	11	29	10	8	27	13	27	5
	$X_{\left(2\right)}^{2}$ = 14.40, *p* < 0.001			$X_{\left(2\right)}^{2}$ = 20.80, *p* < 0.001			$X_{\left(2\right)}^{2}$ = 14.53, *p* < 0.001			$X_{\left(2\right)}^{2}$ = 16.53, *p* < 0.001		
Evaluation 2 (US)	15	11	27	22	8	23	16	5	32	14	31	8
	$X_{\left(2\right)}^{2}$ = 7.85, *p* = 0.02			$X_{\left(2\right)}^{2}$ = 7.96, *p* = 0.02			$X_{\left(2\right)}^{2}$ = 20.87, *p* < 0.001			$X_{\left(2\right)}^{2}$ = 16.11, *p* < 0.001		
**STUDY 3**												
Evaluation 1 (PT)	5	16	25	3	17	26	5	15	26	28	9	7
	$X_{\left(2\right)}^{2}$ = 13.09, *p* = 0.001			$X_{\left(2\right)}^{2}$ = 17.52, *p* < 0.001			$X_{\left(2\right)}^{2}$ = 14.39, *p* = 0.001			$X_{\left(2\right)}^{2}$ = 18.32, *p* < 0.001		
Evaluation 2 (US)	7	25	22	4	26	24	10	22	22	32	12	10
	$X_{\left(2\right)}^{2}$ = 10.33, *p* = 0.006			$X_{\left(2\right)}^{2}$ = 16.44, *p* < 0.001			$X_{\left(2\right)}^{2}$ = 5.33, *p* = 0.07			$X_{\left(2\right)}^{2}$ = 16.44, *p* < 0.001		

*Frequencies of choosing each of the Classification Images (CIs) in forced-choice questions in the three studies are reported. Low, mod, and high refer to the low-adaptation CI, moderate-adaptation CI, and high-adaptation CI, respectively*.

In the first evaluation, we found significant differences on positive adjectives. As expected, Portuguese raters evaluated the low-adaptation CI lower on positive characteristics than the remaining CIs. Pairwise comparisons showed that the only significant mean difference resided between the low-adaptation CI and the moderate-adaptation CI (*p* = 0.02). However, the moderate-adaptation CI was evaluated more positively than the high-adaptation CI. This unpredicted quadratic effect was significant, while the linear effect was not. The results on the composite for negative adjectives and on all forced-choice questions (all *p*s > 0.20) were not significant.

In the second CI evaluation by American raters, we found a significant linear effect on negative adjectives, with the low-adaptation CI evaluated most negatively and the high-adaptation CI evaluated least negatively. A similar linear pattern was found for the forced-choice question “Which person you would never want to meet in a dark empty street at night?” The results on the composite for positive adjectives and on the remaining forced-choice questions (all *p*s > 0.07) were not significant.

The third evaluation by a different sample of Portuguese students replicated the pattern found in the first evaluation. This time, significant quadratic effects were found for both positive adjectives and negative adjectives, with a significant mean difference residing in both cases between the low-adaptation CI and the moderate-adaptation CI (*p*s < 0.003). A similar quadratic pattern was found for one forced-choice question (“Who would you choose to… go to the cinema or a party with”). The remaining forced-choice questions showed significant results with a linear pattern, with the low-adaptation CI chosen least frequently as the person to share a room with or to do university work with, and most frequently as the person not to meet in a dark street [$\chi_{\left(2\right)}^{2}$ = 38.11, *p* < 0.001]. Differences in evaluations on the two dimensions added in this evaluation (“Similar to yourself,” “Similar to a typical Portuguese”) were non-significant (all *p*s > 0.20).

### Discussion of Study 1

Study 1 partially supported our hypothesis. The results of the objective evaluation showed that the low-adaptation image shared no similarities with the moderate and high-adaptation CIs, indicating that representation of locals held by poorly adapted participants indeed differed from those at higher adaptation levels. Consistently with that, while not all differences were significant, the overall pattern of the intersubjective evaluation showed that across the three evaluations, the low-adaptation CI was consistently rated less positively than the moderate and high-adaptation CIs. However, instead of the expected linear effect, we found a quadratic pattern for Portuguese raters: it was the moderate-adaptation CI that had the most positive evaluations, not the high-adaptation CI. This pattern was replicated with a second independent sample of Portuguese raters, ruling out the possibility of the result being spurious.

As this quadratic pattern was limited to raters from the target population (i.e., Portuguese), we hypothesized that high adaptation increases ingroup projection (Wenzel et al., 2007), rendering the CI produced by highly adapted sojourners more similar to the self-stereotype of their home country population than to the self-stereotype of the host country population. However, because this pattern of results was only found in this study and did not reoccur in its replications, we abstain from developing on this hypothesis here. An interested reader may refer to Appendix C in the Supplementary Materials, for more details.

## Study 2

### Methods

Study 2 was designed as a conceptual and direct replication of Study 1 and followed a similar procedure. It was conducted in the following academic year to ensure sample independence.

In Phase I, participating universities were requested to disseminate the online survey only among new international students. To ensure sufficient sample size, we also reached out to expatriate academics from these universities using their public contact details from university websites. A mixed sample of 154 international students (80.5%) and expatriate academics (i.e., post-docs, 19.5%) (41.6% male; 52.6% aged 21–25 years, 22% aged 26–35 years, 10.3% aged over 36 years, and 9.7% aged below 20 years; 78% sojourning in Portugal for 12 months or less; most represented countries: Brazil, 14.9%; Italy, 12.3%; Germany, 9%; 39 other countries with ≤5%) completed an online survey consisting of the same assessment instruments as in Study 1 (direct replication). The dropout rate was 58.4%, which is high but not unusual in online studies (Galesic, 2006).

Similarly as in Study 1, the survey consisted of the 50-trial RCT, measures of cross-cultural adaptation (BPAS, Demes and Geeraert, 2014; SCAS, Ward and Kennedy, 1999) and sociodemographic measures. Additionally, right after the RCT, participants were shown, side by side, the low-, moderate-, and high-adaptation CIs from Study 1 and they were instructed, identically as in the RCT, to choose the CI that looked most like a typical Portuguese person. We assumed that, if the CIs truly corresponded with sojourner representations of locals at different levels of adaptation, participants should choose the CI corresponding with their own adaptation level (conceptual replication). After calculating overall adaptation scores, the sample was split on the 33rd and 66th percentiles into three groups: low adaptation (*N* = 51, *M* = 3.09, *SD* = 0.35), moderate adaptation (*N* = 55, *M* = 3.80, *SD* = 0.16), and high adaptation (*N* = 48, *M* = 4.31, *SD* = 0.20).

In Phase II, the CIs obtained using the procedure of Dotsch et al. (2008; see Study 1) were evaluated objectively, using the same procedure as in Study 1, and intersubjectively, by two rater samples: 46 Portuguese students (48.9% male, mean age: 20.4 years, most represented study domains: management 48.9%, psychology 29.8%) and 53 American raters recruited via MTurk (62.3% male, mean age: 34.3 years). We used an identical survey as employed previously in the third evaluation in Study 1 (i.e., positive and negative adjectives, forced-choice questions).

### Results

#### Conceptual Replication

**Figure 2:**
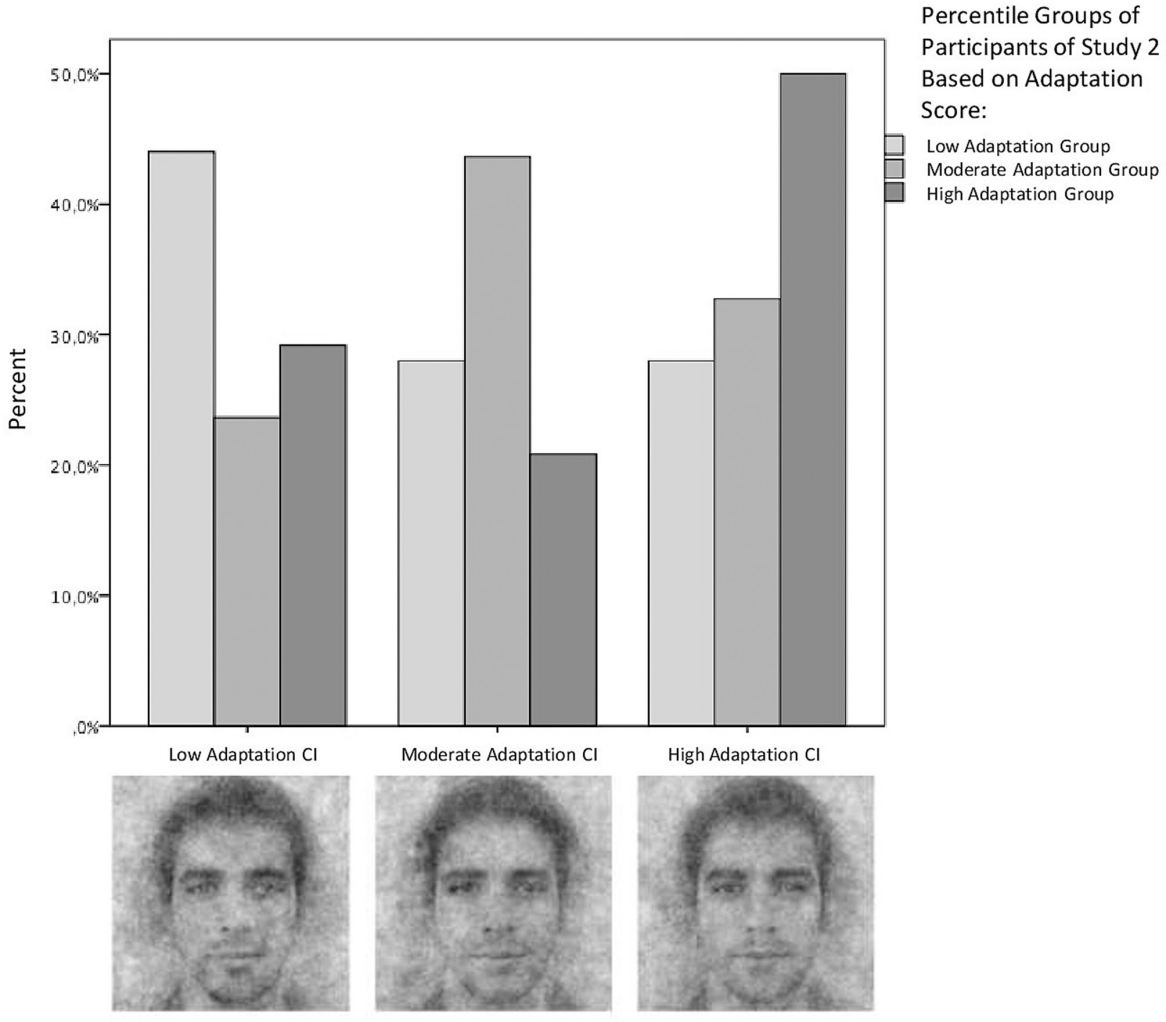
Conceptual replication of Study 1. The bars represent frequencies of choosing the low-, moderate-, and high-adaptation CI (obtained in Study 1; x-axis) as the most prototypical Portuguese face by Study 2 participants with low- (white bars), moderate- (light gray bars), and high- (dark gray bars) adaptation level (sample split on the 33rd and the 66th percentiles).

As expected, the degree of cross-cultural adaptation of participants in Study 2, Phase I was positively correlated with the level of adaptation (1—low, 2—moderate, 3—high) of the CI from Study 1 these participants indicated as most typically Portuguese (Spearman's ρ = 0.18, *p* = 0.03; Figure 2). For an additional analysis, we split the Study 2 sample on the 33rd and the 66th percentile into three adaptation groups to match the three levels of adaptation CIs from Study 1 (low, moderate, high). A chi-squared test conducted with this split sample confirmed that participants chose the CIs corresponding with their own level of adaptation more frequently than the remaining CIs [$\chi_{\left(4\right)}^{2}$ = 11.68, *p* = 0.02]. That is, poorly adapted participants of Study 2 tended to choose the low-adaptation CI from Study 1, moderately adapted participants—the moderate-adaptation CI from Study 1, and highly adapted participants—the high-adaptation CI from Study 1.

#### Direct Replication: Objective Evaluation

Based on the results of Study 1, we expected that the low-adaptation CI will share relatively little physical similarities with the moderate- and high-adaptation CIs. That is, pixel correlations should be weaker between the low-adaptation CI and the remaining CIs and stronger between the moderate- and high-adaptation CIs. In this study, however, the low-adaptation CI showed a weak positive correlation with the moderate-adaptation CI (*r* = 0.09, 95% CI [0.089, 0.097]) and the high-adaptation CI (*r* = 0.17, 95% CI [0.164, 0.171]). Similarly, the moderate-adaptation CI was weakly positively correlated with high-adaptation CI (*r* = 0.17, 95% CI [0.163, 0.171]). In other words, the low-adaptation CI was the least similar to the moderate-adaptation CI, while the degree of similarity between the remaining pairs of images was similar.

#### Direct Replication: Intersubjective Evaluation

The repeated-measures ANOVA testing the differences in the evaluation of the three CIs obtained in Phase I of this study found three significant linear effects (out of four tested; see Table 1): on positive adjectives for both rater samples and for negative adjectives for American raters. The effect on negative adjectives was non-significant for Portuguese raters. The pattern of means shows that Portuguese and American raters rated the high-adaptation CI the highest on positive adjectives and the lowest on negative adjectives, while differentiating less between the low-adaptation CI and moderate-adaptation CI. Pairwise comparisons revealed significant mean differences between the low-adaptation CI and the high-adaptation CI (*p* = 0.01 for both rater samples on positive adjectives, *p* = 0.04 for American raters on negative adjectives), and for American raters also between moderate-adaptation CI and high-adaptation CI (*p* = 0.01 for positive adjectives, *p* = 0.03 for negative adjectives). The unexpected quadratic effect found in Study 1 for Portuguese raters did not replicate.

Moreover, Portuguese raters evaluated the moderate-adaptation CI as the least similar to a typical Portuguese [Greenhouse–Geisser *F*_(1.59, 70.02)_ = 16.00, *p* < 0.001; *M*_low_ =5.67, *SE*_low_ = 0.40, *M*_mod_ = 3.96, *SE*_mod_ = 0.41; *M*_high_ = 5.56, *SE*_high_ = 0.38; quadratic contrast: *F*_(1,44)_ = 22.39, *p* < 0.001; linear contrast: *p* = 0.67], while American raters considered this CI as the least similar to themselves [*F*_(2, 108)_ = 5.94, *p* = 0.004; *M*_low_ = 3.85, *SE*_low_ = 0.28, *M*_mod_ = 3.65, *SE*_mod_ = 0.29; *M*_high_ = 4.54, *SE*_high_ = 0.29; quadratic contrast: *F*_(1, 54)_ = 5.57, *p* = 0.02; linear contrast: *F*_(1, 54)_ = 6.28, *p* = 0.02].

Finally, significant differences were found on forced-choice questions (see Table 2). Overall, the high-adaptation CI was chosen most often for the positive activities and least often as the person they would not like to meet in a dark street. However, both American and Portuguese raters indicated the moderate-adaptation CI most often as the person they would not like to meet in a dark street and American raters least often as their coworker and their roommate. Raters differentiated less between the low-adaptation CI and the moderate-adaptation CI on the remaining items (all *p*s > 0.05).

### Discussion of Study 2

In Study 2, the association between sojourner adaptation and sojourner representation of locals was found again in the conceptual replication. First, we confirmed that the CIs obtained in Study 1 accurately tap into outgroup representations at low, moderate, and high levels of sojourner adaptation. Although the sojourner sample in Study 2 consisted of different participants than those who created the CIs in Study 1, when requested to choose the most prototypical image, these participants still tended to indicate the CI created by a group with a degree of cross-cultural adaptation corresponding with their own. We therefore concluded that the online RCT with 50 randomized trials was sensitive enough to grasp some features of the representation of locals shared by sojourners with a specific level of adaptation but differing between adaptation levels.

The results of the direct replication were less clear. The objective evaluation showed that the low-adaptation CI was the least similar to the moderate-adaptation CI, but this pattern was not found in the intersubjective evaluation. Both Portuguese and American raters consistently attributed more positive traits to the high-adaptation CI, but the moderate-adaptation CI was evaluated similarly, and in some cases even more negatively than the low-adaptation CI. Therefore, we considered these results non-conclusive and we attempted another replication.

## Study 3

### Methods

Study 3 was designed as a direct replication of Study 1 with a more sensitive measure, that is, a long version of RCT with 300 trials produced by participants in the lab instead of online. Besides this modification, the procedure and methods used in this study were identical as in Study 1. Again, we were interested to test if the low-adaptation CI, reflecting the representation of locals at the lowest levels of adaptation to the local culture, would carry significantly more negative valence than the moderate and high-adaptation CIs.

For Phase 1, a mixed sample of 22 international students and migrants was recruited both at the first author's university and using personal contacts (27.3% male, 54.5% aged below 30 years and another 31.8% 30–40 years, 50% sojourning in Portugal for 24 months or less, another 45% between 25 months and 10 years; most represented countries: Poland 31.8%, Brazil 22.7%, Germany 18.2%). The sample size was substantially smaller than in Studies 1 and 2, but because each participant performed the full set of 300 trials, the overall number of trials completed by this sample (~6,600) was comparable to our previous studies (~5,900 in Study 1 and ~7,700 in Study 2). After calculating overall adaptation scores, the sample was split on the 33rd and 66th percentiles into three groups: low adaptation (*N* = 7, *M* = 3.12, *SD* = 0.19), moderate adaptation (*N* = 8, *M* = 3.62, *SD* = 0.11), and high adaptation (*N* = 7, *M* = 4.02, *SD* = 0.19).

In Phase II, CIs produced by the sojourner sample were again evaluated objectively, using the same pixel-wise correlation procedure as in Studies 1 and 2, and intersubjectively, by 46 Portuguese students (32.3% male, mean age: 19.8 years, most represented study areas: management 47.9%, psychology 30.5%) and 53 American raters recruited via MTurk (62.3% male, mean age: 34.3 years). We employed, respectively, the full Portuguese and English version of the survey used previously (third evaluation in Study 1, Study 2).

### Results

#### Objective Evaluation

As expected, the low-adaptation CI showed the smallest correlation with the high-adaptation CI (*r* = 0.26, 95% CI [0.253, 0.260]), and a slightly higher correlation with the moderate-adaptation CI (*r* = 0.30, 95% CI [0.298, 0.305]). The moderate-adaptation CI was moderately positively correlated with high-adaptation CI (*r* = 0.37, 95% CI [0.364, 0.371]). This pattern suggested that the representation of locals held by poorly adapted sojourners is the least similar to that held by highly adapted sojourners and slightly more similar to that held by moderately adapted sojourners.

#### Intersubjective Evaluation

Consistent significant differences in CI evaluation were found across the two rater samples on both positive and negative adjectives. In all cases, means showed significant linear patterns in the expected directions, that is, the high-adaptation CI was evaluated the most positively and the low-adaptation CI the most negatively (see Table 1). The significant mean differences resided between the low-adaptation CI and the high-adaptation CI (all *p*s < 0.05 for both rater samples), with one significant effect between the moderate-adaptation CI and the high-adaptation CI on positive adjectives for Portuguese raters (*p* = 0.04), and one significant effect between the low-adaptation CI and the moderate-adaptation CI on positive adjectives for American raters (*p* = 0.005). Moreover, Portuguese raters evaluated the high-adaptation CI as the most similar to a typical Portuguese [*F*_(2, 86)_ = 7.30, *p* = 0.001; *M*_low_ = 5.07, *SE*_low_ = 0.40; *M*_mod_ = 6.00, *SE*_mod_ = 0.37; *M*_high_ = 6.27, *SE*_high_ = 0.37, linear contrast: *F*_(1, 43)_ = 13.92, *p* = 0.001; quadratic contrast: *p* = 0.27], and American raters as the most similar to themselves [*F*_(2, 106)_ = 4.97, *p* = 0.01; *M*_low_ = 3.31, *SE*_low_ = 0.31; *M*_mod_ = 3.83, *SE*_mod_ = 0.32; *M*_high_ = 4.20, *SE*_high_ = 0.30, linear contrast: *F*_(1, 53)_ = 12.33, *p* < 0.001; quadratic contrast: *p* = 0.78].

Finally, there were significant differences in frequencies of choosing the different CIs in forced-choice questions. Across both rater samples, the low-adaptation CI was the least often indicated as the preferred person to go to the cinema with, to work with, and to cohabitate with (this latter result was non-significant for the American raters) and the most often as the person whom they would not like to meet in a dark street (Table 2). Raters differentiated less between the moderate-adaptation CI and the high-adaptation CI.

### Discussion of Study 3

Study 3 provided further evidence for the link between sojourner adaptation and sojourner representation of locals (*H1*). Both the objective and intersubjective CI evaluations converged in concluding that the low-adaptation CI was the most distinguishable from the high-adaptation CI. Further, the intersubjective evaluation of CIs obtained from the full set of 300 RCT trials was consistent with CI evaluation from Study 1 with 50 randomized trials in that the low-adaptation CI was rated the most negatively. This time, there was a neat linear effect across both composites and individual adjectives (see Appendix B in the Supplementary Materials), indicating that the better the adaptation, the more positively sojourners perceive the local people. This result was found regardless of rater nationality.

## Meta-Analysis

### Methods

Because Studies 1–3 were not equivalent in regard to the shape of the effect of adaptation on sojourner representation of locals, we meta-analyzed the results of these studies to determine between which levels of adaptation the effect resides. First, we meta-analyzed the pixel correlations obtained in the objective evaluations. To do so, we pooled separately the correlations between low-adaptation CI and moderate-adaptation CI in the three studies; the correlations between low-adaptation CI and high-adaptation CI in the three studies; and the correlations between moderate-adaptation CI and high-adaptation CI in the three studies.

Second, we meta-analyzed the differences in the evaluation of each pair of CIs. For all seven evaluations by both Portuguese and American raters, we calculated separate standardized mean differences in CI evaluation (Cohen's *d*) between low and moderate adaptation, moderate and high adaptation, and low and high adaptation. This was done separately for positive adjectives and for negative adjectives. In all cases, the lower adaptation level was taken as the baseline for the calculation so that the effect sizes indicate whether the evaluation is lower (negative sign) or higher (positive sign) at the higher adaptation level than on the lower adaptation level to which it is compared.

In both cases, the effect sizes were then meta-analyzed with the reverse variance weighting method (Lipsey and Wilson, 2001) using *metafor* 2.4-0 package for R (Viechtbauer, 2010). Primary effects were transformed to Fischer's *z* prior to analyses, and results were retransformed from *z* to the original metric (*r, d*). Because the operationalization was virtually identical across all evaluations in the three primary studies, we applied fixed-effects models to perform homogeneity analyses (Hedges and Vevea, 1998). For the intersubjective evaluation, we additionally conducted moderation analyses using rater nationality as a binary moderator.

### Results

Results of both meta-analyses are reported in Table 3. In the pooled objective evaluations, the pixel correlation was non-significant between the low- and moderate-adaptation CIs, and significant but weak between the low- and high-adaptation CIs. Both effects were smaller in size than the significant pixel correlation between the moderate- and high-adaptation CI. This result suggested that across studies, the low-adaptation CIs shared no similarities with the moderate-adaptation CI, and relatively little similarities with the high-adaptation CIs. In all cases, the *Q* statistics were significant, indicating that the effects were heterogeneous. Since *Q* is related to sample size in primary studies, and our primary effects used pixels as the unit of analysis, the unusually large and significant *Q* values may be explained by large pixel *N*s.

**Table 3:** Meta-analysis of effect sizes of CI evaluation across studies 1–3.

	***ES***	***p* (ES)**	***Q***	***p*(Q)**
**OBJECTIVE EVALUATION**				
Moderate vs. low	0.12	0.17	17407.70	<0.001
High vs. low	0.15	0.01	7344.31	<0.001
High vs. moderate	0.27	<0.001	6357.40	<0.001
**INTERSUBJECTIVE EVALUATION**				
**Positive adjectives**				
Moderate vs. low	0.26	<0.001	8.45	0.21
High vs. low	0.37	<0.001	5.22	0.52
High vs. moderate	0.12	0.26	12.14	0.26
**Negative adjectives**				
Moderate vs. low	−0.17	0.02	8.52	0.20
High vs. low	−0.28	<0.001	2.67	0.85
High vs. moderate	−0.10	0.28	8.58	0.20

*ES—effect size. For objective evaluation, ES refers to pooled correlation coefficients (r) between pixel luminosity of CIs corresponding with different levels of adaptation, calculated as fixed effects models, all ks = 3. For intersubjective evaluation, ES refers to pooled standardized mean differences (d) between ratings of CIs corresponding with different levels of adaptation, calculated as fixed-effects models, all ks = 7*.

In the pooled intersubjective evaluations, we found, for both positive and negative adjectives, significant mean differences between the ratings of low and high-adaptation CIs, as well as between the ratings of low and moderate-adaptation CIs. The former effect sizes (low vs. high) were larger than the latter (low vs. moderate) for both composites. The difference between evaluations of moderate- and high-adaptation CIs was not significant for either composite. In all cases, the *Q* statistics were not significant, indicating that the effects are homogenous. In line with our predictions, all effect sizes for positive adjectives had a positive sign, that is, the mean CI evaluation was more positive at higher levels of adaptation. All effect sizes for negative adjectives had negative signs, indicating that the mean CI evaluation less negative at higher levels of adaptation. No moderating effects of rater nationality were found (all between-groups *p*s > 0.20), indicating that differences in the evaluation of the different CIs do not differ between American and Portuguese raters.

### Discussion

The meta-analysis consolidated and reinforced our findings by showing that, all CI evaluations taken together, the degree of sojourner adaptation and the valence of sojourner representations of locals are interrelated. Across the three studies, the objective and intersubjective evaluations showed the same pattern: it was the CI corresponding with the representation of locals at the lowest levels of adaptation that stood out. Independently of rater nationality, the significant difference resided between low adaptation level and the remaining levels, suggesting that poorly adapted sojourners hold a relatively negative representation of the host-national outgroup. However, because there was significant pixel similarity between moderate and high adaptation, and a small and statistically insignificant mean difference in intersubjective ratings, these results seem to indicate that the empirical link between adaptation and valence of the representation of locals is not equally strong at all adaptation levels.

## General Discussion

The three studies reported above show that sojourner adaptation is reflected in the valence of sojourner representations of the host national outgroup: poor adaptation at the Affect and Behavior level is correlated with negative visual representations of locals. We assume that these results indicate the hypothesized Cognition level of adaptation: cross-cultural adaptation does manifest itself not only in increased well-being and increased adequacy of behaviors within the host culture but also in the way we think of typical members of the majority host culture. In this regard, consistent results were obtained using three different methods (reduced RCT, full RCT, conceptual replication) with three independent samples of sojourners and seven samples of raters of two nationalities. These findings are in line both with the adaptation literature associating poor adaptation with high intergroup tension (Wilson et al., 2013) and with the intergroup literature associating high intergroup tension with negative representations of outgroups (Dotsch et al., 2008).

Interestingly, the difference in valence of outgroup representations seems to reside between poorly adapted sojourner groups and the remaining sojourners. The low-adaptation CI tended to be evaluated more negatively than the moderate and high-adaptation CIs, and the statistical significance of this difference was supported by the final meta-analysis. The meta-analysis found no difference between the moderate and high-adaptation CIs, suggesting that representations of locals at these levels are similar in valence.

One possible reason could be that at low levels of adaptation, when host culture awareness is low and behaviors of locals seem incomprehensible, threat is at stake; at intermediate and high levels, when one has learned more about the host culture and it has partially lost its threatening features, it is more challenges about finding one's way around in the host society and making contacts (van der Zee and van Oudenhoven, 2013). It seems plausible, therefore, that it is at this very first culture shock level that the representations of locals are negative. An alternative explanation could be that the advancing adaptation might approach an optimal representation of locals, rather than mechanistically render it more and more positive. In this case, the differences between moderate- and high-adaptation level may not be captured by valence. Although determining what such an optimal representation should look like may be difficult or impossible, future research may test this hypothesis by using more sophisticated dependent variables to capture how adaptive sojourner representations are (e.g., flexibility or context sensitivity).

Lastly, future research may investigate the content of sojourner representations of locals in greater detail. Although we used a diverse set of adjectives derived from various theoretical sources, including warmth and competence, group attractiveness, and conflict-related aspects potentially relevant to adaptation, all items measuring these aspects showed very similar patterns at the different levels of adaptation, and the only feature that seemed to make a difference was their valence. However, this result does not necessarily mean that stereotype content remains stable as adaptation progresses. Since our studies were not preceded by a prototype analysis, there is some possibility that, in our attempts to diversify the adjectives to cover all adaptation-related aspects, we might have overlooked some relevant ones. Future studies may use prototype analysis to obtain new insights into links between stereotype content and adaptation.

### Methodological Remarks

In the above studies, we adopted a well-known two-phase reverse correlation procedure (Dotsch et al., 2008). The drawback of this procedure is that it may lead to increased rates of Type I error because the transition from the CI creation phase to the CI rating phase does not take into account that the variation occurring in the group CIs is added to the variation occurring in the ratings task. As a result, test results may be significant even if differences between the CIs are random (Cone et al., 2020). While no empirical study can entirely rule out that its findings are due to random error, we consider that in our studies the probability that the differences between the CIs were random is very low as we addressed this issue in two ways. First, we computed pixel correlations (see Oliveira et al., 2019) to assess whether the degree of physical similarity between the different CIs was consistent with (a) our theoretical predictions, which would be unlikely if the physical differences between CIs were random, and (b) with the ratings, which would be unlikely if the raters' responses to these differences were random. Both (a) and (b) were met in Studies 1 and 3, but less so in Study 2. However, the final meta-analysis showed that all results taken together, even including the inconclusive effects from Study 2, the pattern of pixel correlations converged with both the predictions and CI ratings, suggesting that the physical differences between CIs were not random and the raters' responses to these physical differences were not random.

Second, we conducted four replications in which we obtained similar patterns of results, which would be unlikely if our results were indeed due to Type 1 error because significant findings due to such error should occur with equal probability in the hypothesized direction (low-adaptation CI as the most negatively rated) and in the opposite direction (low-adaptation CI as the most positively rated). Moreover, we included a conceptual replication (Study 2) where we reversed the process: we showed our participants CIs created by other sojourners and asked them to choose the most prototypically Portuguese one. If the differences between the CIs were random, participants would choose between them at random. This was most probably not the case; participants tended to choose the image that matched their own adaptation level, which again shows that the differences between the CIs were not random. Taken together, the convergence of pixel correlations with independent judges' ratings and the conceptual replication clearly indicate that the differences between the CIs were meaningful, which sharply reduces the probability of inflated Type I error in our studies.

## Conclusion

The current set of studies offers evidence that the valence of sojourner perceptions of locals is associated with sojourner degree of adaptation to living among these locals. This association is unlikely to be an artifact coming from social desirability or experimenter effects; first, because our method tapped into implicit associations and only partially relied on sojourner self-reports, and second, because the raters had no indication where the CIs came from and what they represented. Therefore, we believe our findings reveal the social-cognitive component of adaptation, the neglected C of the ABC model of cross-cultural adaptation (Ward et al., 2001). They encourage further theoretical elaboration of the concept and open a new promising avenue in adaptation research.

Moreover, our results point to the inherent intergroup nature of cross-cultural adaptation, a perspective that, if applied in future research, may help in grasping the phenomenon of adaptation in its full complexity. This perspective is also crucial from an applied point of view. If we aim at a harmonious coexistence of different cultural groups within diverse societies, the link between adaptation and intergroup relations has to be taken into account. It implies that immigration policies and intervention programs supporting cross-cultural adaptation of immigrants and sojourners are beneficial not only for their target groups but also for the society as a whole: they contribute to improved relations between these newcomers and the local people, to decreased intergroup tension and to a lowered risk of conflict. These benefits extend to any member of the host society and make investing in cross-cultural adaptation doubly worthwhile.

## Data Availability Statement

The raw data supporting the conclusions of this article will be made available by the authors, without undue reservation.

## Ethics Statement

Ethical review and approval was not required for the study on human participants in accordance with the local legislation and institutional requirements. The patients/participants provided their written informed consent to participate in this study.

## Author Contributions

KB, SW, and KZ designed the studies, drafted the paper, and provided critical revisions. KB collected the data and conducted the analyses. SW and KZ provided critical feedback. All authors contributed to the article and approved the submitted version.

## Conflict of Interest

The authors declare that the research was conducted in the absence of any commercial or financial relationships that could be construed as a potential conflict of interest.

## Footnotes

^1^We assume that the drop in Cronbach's alpha in Study 3 had to do with alpha's sensitivity to sample size. Recent simulations show that about 31 participants are needed to reach an alpha of 0.70 with a 12-item measure (Bujang et al., 2018), which could explain why the standard thresholds for alpha were not achieved in Study 3 (*N* = 22).

^2^Oliveira et al. (2019) proposed masking the irrelevant parts of the image before calculating the correlations. However, in our case the impact of masking the images on the results was negligible across all studies; therefore, all results refer to unmasked images.

## Supplementary Material

Data Sheet 1 Appendix A and B.

Data Sheet 2 Appendix C.
