# Supplementary material for: The Neglected C of Intercultural Relations. Cross-Cultural Adaptation Shapes Sojourner Representations of Locals
Source: Front Psychol. 2021 Mar 23;12:611630. doi: 10.3389/fpsyg.2021.611630 (PMC8021927; doi:10.3389/fpsyg.2021.611630)
Supplement: Data Sheet 2 — Appendix C. [file Data_Sheet_2.doc]

The Neglected C of Intercultural Relations. Cross-Cultural Adaptation Shapes Sojourner Representations of Locals

Supplementary Materials, Appendix C

**Projection Phenomena in Cross-Cultural Adaptation**

The overall pattern of results of the ratings by the first rater sample in Study 1was quadratic instead of showing the expected linear pattern: it was the moderate adaptation CI that had the most positive evaluations, not the high adaptation CI. As an explanation for this unexpected pattern we suspected that the reason could rest in the fact that the raters were members of the RCT target population (i.e., Portuguese) and that the evaluations of the high adaptation CI was less positive than expected due to social projection (Clement & Krueger, 2002) and/or ingroup projection (Mummendey & Wenzel, 1999) shown by highly adapted sojourners. The reasoning is the following. Best adaptation tends to be achieved by sojourners who endorse and identify with both the host and the home culture (acculturation strategy of integration, Berry, 1997, 2005; or biculturalism, see Nguyen & Benet-Martinez, 2013, for a meta-analysis). This may suggest that highly adapted sojourners may, rather than see locals as a discrete outgroup, perceive them as a group in which they see themselves included, that is, either as an ingroup or as a superordinate category (which is also an ingroup but on a higher level of inclusiveness, e.g., Europeans, humans, inhabitants of Portugal).

Such recategorization may affect projection processes (cf. Clement & Krueger, 2002; Otten, 2005). While people do not tend to project their own individual features (social projection) and/or distinctive features of their group (ingroup projection) to outgroups, they do project them to ingroups and superordinate categories, respectively. Moreover, such ingroups and superordinate categories tend to be evaluated positively (Wenzel, Mummendey & Waldzus, 2007; Waldzus & Mummendey, 2004; Waldzus et al., 2003). Therefore, if highly adapted sojourners indeed see locals as members of a common superordinate category, their representations should not only be more positive, but also influenced by social and/or ingroup projection. Projection phenomena are, in turn, reflected in visual representations of group prototypes (Imhoff et al., 2011; Imhoff et al., 2013; Imhoff & Dotsch, 2013). For example, in RCTs asking which of two stimulus faces looks more typically European, Germans produce more German looking CIs than Portuguese do (Imhoff et al., 2011). By analogy, a highly adapted German sojourner should see a Portuguese local person as more German looking than a poorly adapted German sojourner.

Therefore, while visual representations of locals held by highly adapted sojourners should be the most positive in their valence, we may also expect them to be contaminated by projection. Highly adapted sojourners are likely to overestimate the similarity of local people to themselves and/or their primary ingroup, that is, their co-nationals. In this sense, their visual representation of locals may approximate itself to the self-stereotype of the sojourner’s primary ingroup (i.e., co-nationals) and thereby drift further away from the locals’ self-stereotype.

In operational terms, one could expect that the CIs produced by highly adapted sojourners would be more similar to the self-stereotype of their home country population and therefore less similar to the self-stereotype of the host country population (Portuguese) than the CIs produced by poorly adapted sojourners. Portuguese raters, in turn, should respond less positively to a CI that has less similarity with their self-stereotype, and more positively to the CI that corresponds best with their self-stereotype. That is, the high adaptation CI should obtain less positive evaluations from Portuguese raters than the moderate adaptation CI because the latter CI should be less affected by projection.

We tested this projection explanation by developing the hypothesis that ratings of CIs produced by highly adapted sojourners should only be affected if raters are members of the target population (i.e., Portuguese) but not if they are members of an unrelated population (*H2*). That is, raters from an unrelated national group should only respond to valence and not to projection. Such a third country rater sample should evaluate the high adaptation CI better than the moderate adaptation CI.

We tested this hypothesis by having the CIs produced in Study 1 rated again by two additional rater samples, one sample of Portuguese raters and one sample of US-American raters recruited via MTurk. We predicted that Portuguese raters should replicate the quadratic pattern produced by the first sample of Portuguese raters. In contrast, for the US-American raters we predicted that they produce the linear pattern predicted by our original hypothesis, because raters of a nationality different than Portuguese, to whom the Portuguese self-stereotype is irrelevant, should not be influenced by the projection effect reflected in the high adaptation CI. Instead, they should only respond to the valence of the presented images. For such raters, CI evaluation should therefore give linear results, with the high adaptation CI evaluated the most positively.

Results showed the expected pattern. We replicated the quadratic pattern for Portuguese raters while evaluations on negative attributes by American raters followed indeed a linear pattern (see Table 1 in the main text), which seemed to sustain our reasoning that high adaptation goes in pair with social or ingroup projection phenomena. Overall, Study 1 supported both the valence effect (*H1*; because low adaptation CIs were evaluated less positively than high adaptation CIs) and the projection effect (*H2*).

However, this pattern of results did not replicate in Study 2 and 3. In Study 2, against our predictions, the moderate adaptation CI was considered the least similar to the Portuguese self-stereotype and tended to obtain the most negative ratings from both Portuguese and American raters (see Table 1 in the main text). In Study 3, there was a neat linear effect across both composites (see Table 1 in the main text) and individual adjectives (see supplementary materials, Appendix B), indicating that the better the adaptation, the more positive the representation of local people. Contrary to *H2*, this result was found regardless of rater nationality, further challenging the projection effect. Finally, our projection hypothesis (*H2*) would imply that the overall effect of CI evaluation should be moderated by rater nationality: Portuguese raters should prefer the moderate adaptation CI and American raters should prefer the high adaptation CI. No such moderation effect was found in the final meta-analysis of the three studies.

To sum up, despite the promising initial results, we failed to find further evidence for projection phenomena related to high adaptation. One possible explanation is that there is no projection and the findings of Study 1 were spurious. However, we may consider an alternative possibility, that is, moderation by some unknown characteristics of the sojourners who produced the CIs. Because in Study 1 (but only in Study 1) the quadratic effect was replicated with a different sample of Portuguese raters, we can assume this pattern had to do with the CIs themselves, or with the features of the sojourner sample behind them, rather than with the features of the rater sample. The absence of such quadratic pattern in Study 2 and 3 could mean that, for some unknown reasons, only the first sojourner sample was prone to projection while the remaining two samples were not.

Various moderating factors could explain this discrepancy, although none of them seems to have been captured by our studies. None of the sociodemographic characteristics and control variables measured by us seemed to differentiate the first sojourner sample enough to be considered as a potential moderator. A further theoretical reflection is needed to propose other plausible moderators of this effect to be tested in future studies.

References

Berry, J. W. (1997). Immigration, acculturation, and adaptation. *Applied Psychology, 46*, 5-34.

Berry, J. W. (2005). Acculturation: Living successfully in two cultures. *International Journal of Intercultural Relations, 29*(6), 697-712.

Clement, R. W., & Krueger, J. (2002). Social categorization moderates social projection. *Journal of Experimental Social Psychology*, *38*(3), 219-231.

Dotsch, R., & Todorov, A. (2012). Reverse correlating social face perception. *Social Psychological and Personality Science, 3*(5), 562-571.

Dotsch, R., Wigboldus, D. H., Langner, O., & van Knippenberg, A. (2008). Ethnic Out-Group Faces Are Biased in the Prejudiced Mind. *Psychological Science, 19*(10), 978-980.

Imhoff, R., & Dotsch, R. (2013). Do we look like me or like us? Visual projection as self-or ingroup-projection. *Social Cognition, 31*(6), 806-816.

Imhoff, R., Dotsch, R., Bianchi, M., Banse, R., & Wigboldus, D. H. (2011). Facing Europe: Visualizing Spontaneous In-Group Projection. *Psychological Science, 22*(12), 1583-1590.

Imhoff, R., Woelki, J., Hanke, S., & Dotsch, R. (2013). Warmth and competence in your face! Visual encoding of stereotype content. *Frontiers in Psychology, 4*, 386.

Mummendey, A., & Wenzel, M. (1999). Social discrimination and tolerance in intergroup relations: Reactions to intergroup difference. *Personality and Social Psychology Review*, *3*, 158–174.

Nguyen, A. M. D., & Benet-Martínez, V. (2013). Biculturalism and adjustment: A meta-analysis. *Journal of Cross-Cultural Psychology*, *44*(1), 122-159.

Otten, S. (2005). The in-group as part of the self: Reconsidering the link between social categorization, in-group favoritism and the self-concept. In A. Alicke, D. Dunning, & J. Krueger (Eds.), *The self in social judgment* (pp. 241–265). Philadelphia: Psychology Press.

Waldzus, S., & Mummendey, A. (2004). Inclusion in A Superordinate Category, In-Group Prototypicality, And Attitudes Towards Out-Groups. *Journal of Experimental Social Psychology*, *40*(4), 466-477.

Waldzus, S., Mummendey, A., Wenzel, M., & Weber, U. (2003). Towards tolerance: Representations of superordinate categories and perceived ingroup prototypicality. *Journal of Experimental Social Psychology*, *39*(1), 31-47.

Wenzel, M., Mummendey, A., & Waldzus, S. (2007). Superordinate Identities and Intergroup Conflict: The Ingroup Projection Model. *European Review of Social Psychology, 18*(1), 331-372.
